# Comprehensive Review of Adipose Stem Cells and Their Implication in Distraction Osteogenesis and Bone Regeneration

**DOI:** 10.1155/2015/842975

**Published:** 2015-09-13

**Authors:** Mina W. Morcos, Hadil Al-Jallad, Reggie Hamdy

**Affiliations:** ^1^Division of Paediatric Orthopaedic Surgery, Shriners Hospital for Children, Montreal Children Hospital, McGill University, 1529 Cedar Avenue, Montreal, QC, Canada H3G 1A6; ^2^Department of Experimental Surgery, McGill University, 1650 Cedar Avenue, Montreal, QC, Canada H3G 1A4; ^3^Orthopaedics Department, Shriners Hospital for Children, 1529 Cedar Avenue, Montreal, QC, Canada H3G 1A6; ^4^McGill University Health Centre, Shriners Hospital for Children, Montreal Children Hospital, 1529 Cedar Avenue, Montreal, QC, Canada H3G 1A6

## Abstract

Bone is one of the most dynamic tissues in the human body that can heal following injury without leaving a scar. However, in instances of extensive bone loss, this intrinsic capacity of bone to heal may not be sufficient and external intervention becomes necessary. Several techniques are available to address this problem, including autogenous bone grafts and allografts. However, all these techniques have their own limitations. An alternative method is the technique of distraction osteogenesis, where gradual and controlled distraction of two bony segments after osteotomy leads to induction of new bone formation. Although distraction osteogenesis usually gives satisfactory results, its major limitation is the prolonged duration of time required before the external fixator is removed, which may lead to numerous complications. Numerous methods to accelerate bone formation in the context of distraction osteogenesis have been reported. A viable alternative to autogenous bone grafts for a source of osteogenic cells is mesenchymal stem cells from bone marrow. However, there are certain problems with bone marrow aspirate. Hence, scientists have investigated other sources for mesenchymal stem cells, specifically adipose tissue, which has been shown to be an excellent source of mesenchymal stem cells. In this paper, the potential use of adipose stem cells to stimulate bone formation is discussed.

## 1. Introduction

Orthopaedic surgeons are always confronted with cases of extensive bone loss—known as critical size defects (CSDs)—where outside intervention is necessary for healing to occur. These large defects may be secondary to trauma, postresection of tumors, or postdebridement of infections. The goal in such cases is to align the bone segments together, facilitate union, obtain equal limb length, and restore the function of the traumatized site [1–4]. Various surgical techniques have been used to address these CSDs, including the gold standard autogenous bone grafts, allografts, various bone grafts substitutes, and vascularized and nonvascularized bone grafts [1, 3, 5–9]. However, all these techniques do have multiple limitations [7, 8]. In 1905, an Italian orthopaedic surgeon called Codivilla performed the first lengthening procedure, where he applied skeletal traction through a calcaneal pin followed with an osteotomy of the femur. This proved that limb lengthening could be achieved without jeopardizing the regeneration of muscles and soft tissues [10]. However, the idea of using traction to promote bone regeneration to treat bone defect only gained popularity when a Russian surgeon, Gavriil Ilizarov, developed a revolutionary surgical technique for distraction osteogenesis (DO). He implemented the law of tension stress and pioneered the biological principles of bone and soft tissue regeneration under slow and gradual distraction [11–13].

## 2. Distraction Osteogenesis

**Figure 1:**
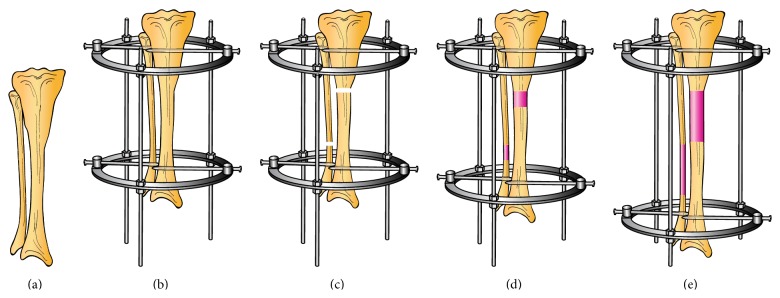
Schematic representation of distraction osteogenesis technique. (a) The tibial bone that needs lengthening. (b) Application of circular external fixator. (c) Tibial and fibular osteotomy. (d) Distraction phase and new bone formation. (e) Consolidation phase.

**Figure 2:**
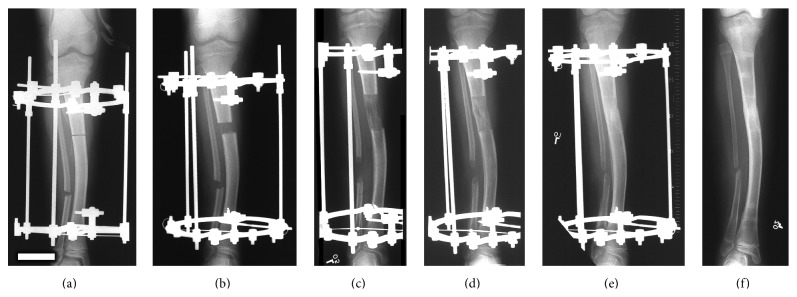
Lengthening of short tibia showing various phases of the distraction process. (a) Application of the fixator and osteotomy of the tibia. (b) Start of distraction. (c) End of distraction. (d and e) Consolidation phase, without any distraction until bone in the distracted gap consolidates. (f) Removal of the fixator (bar scale = 5 cm).

DO is a controlled surgical technique in which the intrinsic capacity of bone to heal and regenerate spontaneously is being used to lengthen short bones (Figure 1(a)) or to replace large segments of bone. DO consists of applying an external fixator to the affected bone in order to immobilize the proximal and distal ends of the bone providing stability (Figure 1(b)), followed by low energy osteotomy to divide the bone into two segments (Figure 1(c)). Latency phase that varies between 5 and 10 days, according to the age, is required following the osteotomy to allow the formation and organization of a hematoma and the recruitment of inflammatory cells and mesenchymal stem cells (MSCs) [14–16]. Following this phase, distraction is started and the two bone segments are gradually distracted at a rate of 1.0 mm/day and a rhythm of 0.25 mm every 6 hours until the required lengthening is attained (Figure 1(d)). Distracting more than 2 mm/day may lead to poor or delayed regenerated bone formation while distracting at a lower rate such as 0.5 mm/day leads to premature consolidation [12, 16]. The final phase is referred to as the consolidation phase in which the distraction is ceased and the newly formed bone is allowed to mature and consolidate before the removal of the device (Figure 1(e)). This is the longest phase in DO, requiring about one month for each centimeter lengthened [16]. Finally, the external fixator is removed once the newly formed bone is well consolidated and deemed strong enough to withstand external forces [11, 17, 18].  Figure 2 represents the different phases of DO.

## 3. Types of Bone Formation Involved in Distraction Osteogenesis

The exact mechanism and type of bone formation in DO are still being debated; however many factors have been identified to play a major role in determining which type of bone formation will predominate including stability of the fixator, rate and rhythm of distractions, and the vascularity of the surrounding tissues [14].

Three types of ossification have been described to take place during new bone formation in DO. Endochondral bone formation usually occurs at early stages of DO and it occurs external to the periosteum immediately adjacent to the fracture site where there is mechanical instability, whereas intramembranous ossification occurs at a later stage [5, 19–22]. In our lab, we were able to demonstrate the presence of both types of ossification in several animal models of DO [23, 24]. Last type is called transchondroid bone formation where chondroid bone is formed directly by chondrocyte-like cells that change gradually from fibrous tissue to bone [5, 22].

## 4. The Molecular and Cellular Events in DO

**Figure 3:**
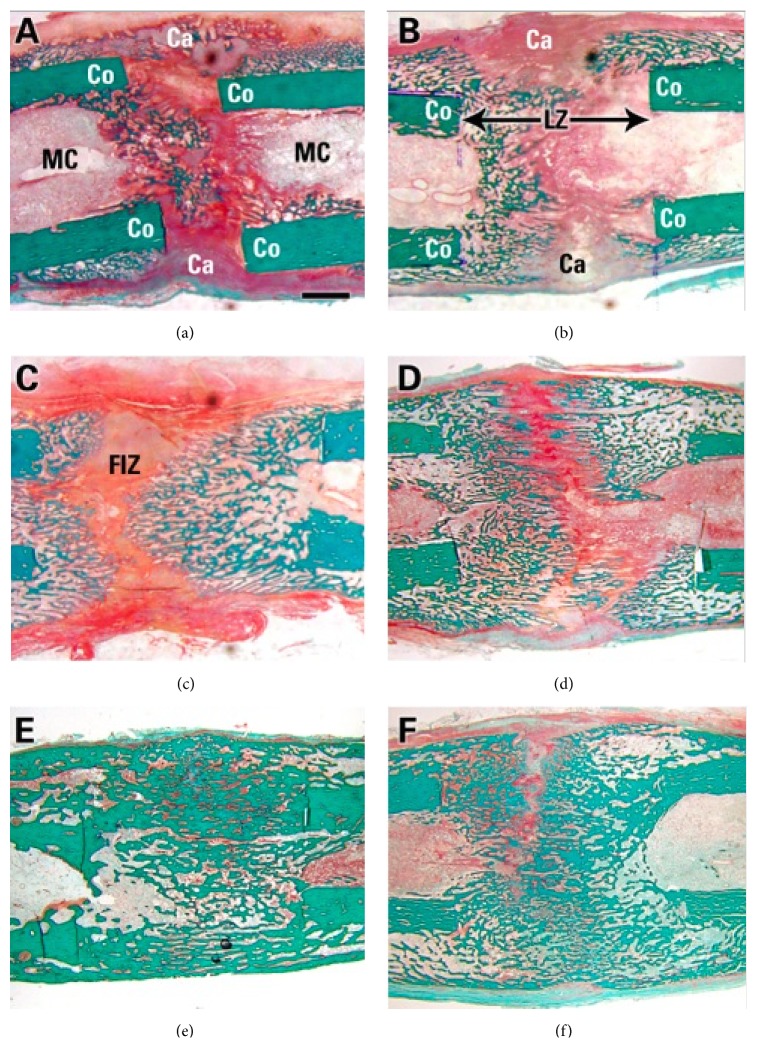
Histological changes using trichrome staining, in a rabbit DO model during distraction osteogenesis of the tibia. (a) to (c) represent the cellular change that happen during the distraction phase while (d) to (f) represent the cellular change that happen during the consolidation phase. Co: cortex; LZ: lengthened zone; Ca: callus; FIZ: fibrous interzone (bar scale = 2 mm).

During the latency phase there is an inflammatory response that leads to the recruitment of MSCs and the secretion of proinflammatory cytokines (interleukin-1, interleukin-6), different growth factors such as transforming growth factor-beta (TGF-*β*), bone morphogenetic proteins (BMPs), and angiogenic factors [5, 21, 25]. These factors are responsible for recruiting MSCs and promoting them to differentiation and proliferation into chondrocytes and osteocytes. Differentiation of these cells is associated with increase expression of type 1 collagen and alkaline phosphatase [5, 8, 26]. During the distraction phase there is gradual differentiation into fibrous and fibrocartilaginous tissue that will be organized in a parallel pattern to the distraction forces [23]. This new bone starts forming from the osteotomy cuts towards the center forming a fibrous radiolucent interzone between the edges of the bone segments (Figure 3) [20, 23, 27, 28]. The fibroblast cells and collagen fibers are arranged longitudinally along the axis of distraction. In addition, during this phase all the surrounding soft tissues are lengthened at the same time with the formation of the new bone and the formation of new blood vessels with intense angiogenesis, neoangiogenesis, and recruitment of osteoblasts [21, 29]. DO was shown to be a vascular dependent process where multiple neoangiogenesis and angiogenesis factors are found within the distracted zone, including vascular endothelial growth factor (VEGF) and angiopoietin factors [21, 30–32]. These vascular processes occur mainly during the latency and distraction phase and then decrease gradually over time. Furthermore, as distraction progresses there is a progressive increase into the deposition of osteoblast along the periosteum and in the distracted gap that is regulated with the mechanical strain applied to the callus. New bone formation during DO is a perfect example of mechanotransduction, where the mechanical forces are converted into molecular and biochemical signals that will activate and regulate multiple cellular events such as neovascular proliferation [21], differentiation, proliferation, and secretory function of various cells including various cytokines, BMPs, extracellular matrix protein, growth factors, and even MSCs [30, 33–36]. This will help maintain the delicate balance between bone formation and bone resorption [14, 37]. We and others have shown that the expression of various cytokines and growth factors is upregulated when exposed to the distraction forces while the same factors are downregulated when distraction is ceased. The expression of these various factors, including BMPs, Wnt signaling, insulin growth factor (IGF), fibroblast growth factor (FGF), TGF-*β*, platelet-derived growth factor (PDGF), and VEGF, is directly related to osteogenesis and chondrogenesis [35, 38–50].

## 5. Advantages and Clinical Significant of DO

Ilizarov method of DO has gained much popularity throughout the years because of its ability to produce new bone between two vascularized surfaces created by osteotomy and followed by gradual distraction [51]. It is considered to be a unique tissue engineering technique since it can spontaneously regenerate vascularized bone of the same micro- and macrostructure of the native bone* in vivo* without the need for bone grafts. Moreover, there are simultaneous regeneration and lengthening of the surrounding soft tissues [10]. Donor site morbidity as seen in autologous grafting is absent. DO is considered the best* in vivo* bone tissue engineering technique [18, 51–53].

## 6. Disadvantages and Complications of DO

The main disadvantage of DO is the long time the external fixator has to stay in place [54]. For every centimeter lengthened, the fixator has to be kept in place for about a month. For example, a child undergoing a 5.0 cm lengthening will require the fixator to be kept in place for about 5 months. This prolonged period of time can lead to multiple complications such as pin-tract infections, broken wires, and joint impairment. Moreover, applying the fixator for a long time can be cumbersome for patients and their families psychologically, socially, and financially [54–56]. Since distraction phase cannot be accelerated as this will lead to poor bone regeneration, then the question arises; how can we accelerate the consolidation phase in order to be able to remove the external fixator at an earlier time?

## 7. Modalities Used in an Attempt to Accelerate DO

Multiple modalities to accelerate bone formation in the context of DO have been described including the application of external biophysical stimuli (i.e., mechanical loading [57, 58], vibration [59], electrical stimulation [60, 61], extracorporeal shock wave [62, 63], and low-energy pulsed ultrasound [64–66]), administration of systemic agents (i.e., sclerostin [67], calcitonin [68], bisphosphonates [69, 70], and prostaglandin E2 [71]), and local agents (i.e., growth factors [72, 73], BMPs [42, 44, 50, 74], scaffolds [39, 75], nanoparticles [76, 77], and osteogenic cells including autologous bone graft and MSCs [78, 79]). In the following sections osteogenic cells, scaffolds, and growth factors will be briefly discussed and then a comprehensive review of MSCs with an emphasis on adipose tissue derived mesenchymal stem cells (ASCs) and their application in bone regeneration and DO will be done.

## 8. Osteogenic Cells

The first important element for bone regeneration is to have a source that will provide viable cells that can differentiate and proliferate into osteogenic cells. For the longest time autologous bone graft has been used and considered as the gold standard material for bone regeneration in orthopedic surgery [8, 80]. Autologous bone is usually harvested from the anterior and posterior iliac crests of the pelvis. It can also be harvested as vascularized bone graft containing an internal vascular network in order to restore a significant bone defect [9, 81, 82] or tricortical graft for structural support [83]. Autologous bone graft possesses many advantages including significant decrease in union time, high union rates, and the ability to restore critical size defects. Moreover, since it uses patient's own tissue, there is a reduction in the risk of immunoreactions and transmission of infections. Finally it possesses osteogenic, osteoconductive, and osteoinductive capacities through the bone growing cells, different proteins, and growth factors that it contains while providing a scaffold for the new bone to grow within, respectively [83]. Although autologous bone graft is a safe and effective way to provide bone cells still it has multiple limitations including donor site morbidity, limited cells quantity, requirement of a second surgical procedure with frequent consequences of pain, and complications [84–86].

An alternative method to harvest autologous bone graft is reamer irrigation aspiration (RIA) system [87]. RIA is an intramedullary reaming system that provides continuous irrigation and aspiration during intramedullary reaming. It was originally designed to decrease the adverse effects of reaming long bone fractures by collecting the reaming material which contains a significant number of osteogenic cells [88]. This provides a large volume of corticocancellous bone material that can be used as autologous bone graft. This is usually harvested from the femur [89]. This technique provides a large volume of autologous bone graft that corresponds to the bone graft obtained from both the anterior and posterior iliac crest [90]; however it still has similar limitations regarding the need for a second operation and the limited quantity of cells that can be provided.

Another methods would be allograft bone that is available in different preparation. However, allograft bone lacks osteogenic capacity as it does not contain living bone cells; therefore it is not considered to be a good source for osteogenic cells. Moreover it carries the risk of disease transmission and immunogenic responses [91]. Therefore it does not fit well with the previously mentioned concepts.

Since both autograft and allograft have restrictions, scientists have investigated the used of MSCs as mentioned above. MSCs are able to differentiate and proliferate into osteogenic cells under the appropriate molecular signals. MSCs have been found in multiple tissues including bone marrow and adipose tissues [92–95]. MSCs will be discussed in more detail in the following sections.

## 9. Scaffolds

Scaffolds are three-dimensional porous structures that need to be biocompatible and biodegradable, producing low immunogenic and antigenic reactions, and have mechanical properties similar to load-bearing bone. They provide support to the attached cells and provide space for it to proliferate (Figure 5) [96]. Scaffolds can be divided into different classes according to the materials used. These include natural polymer (e.g., collagen, fibrin, alginate, and chitosan), synthetic polymers (e.g., polylactic acid (PLA) and polyglycolic acid (PLGA)), and inorganic materials (e.g., calcium phosphates, silicate glasses, hydroxyapatite (HA), ceramics, and calcium sulfate) [3, 75, 97, 98]. These materials have limitations when used on their own including brittleness (e.g., inorganic materials), inflammatory response (e.g., synthetic polymers), and immunogenicity (e.g., natural polymers), which led scientists to investigate nanocomposite scaffolds for their advantages [99]. These advantages include the ability to penetrate deep in tissues without causing damage to the surrounding cells, unique size-scale, large surface to volume ratio, the ability to provide an appropriate vehicle to direct stem cells into specific lineages, the ability to mimic the composition of the natural bone, low immunogenicity, and the ability to enhance the mechanical properties of inorganic materials [100–102]. They can be used as two-dimensional (2D) scaffolds where it covers the implants promoting bone regeneration by allowing cell attachment and tissues to grow on the implant surfaces only or three-dimensional (3D) scaffolds that allow cells to grow on the surface and within it too [103, 104]. 3D scaffolds were found to improve proliferation, differentiation, and activity of cells that grow within them [105]. Environment that mimics the conditions found in the bone microenvironments will help the differentiation of MSCs into osteogenic lineage. This will provide the necessary signals to help MSCs to differentiate and proliferate into the osteogenic lineage in order to repair and regenerate bone. Using the nanocomposite scaffolds technology can provide this environment by providing a controlled environment to the implanted cells, help regulate delivery of nutrients and removal of waste, and enhance the direct differentiation of ASCs into the osteogenic lineage. Bone is composite of a very complex extracellular matrix that helps in maintaining the structural integrity of bone and influencing the differentiation and proliferation of stem cells into the osteogenic lineage [106, 107]. Extracellular matrix consists of organic component, composed mainly of mineralized calcium in the form of HA and a mixture of other inorganic salts [106, 108, 109], and inorganic component, primary composed of 95% type I collagen and other noncollagenous proteins [110, 111]. Therefore, it is very important to be able to provide the appropriate nanoparticle composite to induce and enhance the differentiation of ASCs into osteogenic lineage. Few studies have been done looking at different materials and their effect on ASCs. Lu studied the effect of 3D-nanocomposite scaffold composed of biphasic calcium phosphates (BCP) coated with a nanocomposite layer of polycaprolactone (PCL) and HA-nanoparticles and their effect on osteogenic differentiation of ASCs. Lu et al. were able to show that ASCs on BCP/PCL-nHA had earlier osteogenic differentiation compared with control [112]. Ghorbani and his colleague looked at a slightly different nanoparticle composite where they used PCL/Chitosan (Ch)/zinc-doped HA (nZnHA) nanocomposite with ASCs. They were able to demonstrate an increase in ASCs attachment to the nanocomposite and more important significant increase in the proliferation rate of ASCs compared with control [113]. In another study by McCullen et al., they fabricated scaffolds consisting of B-tricalcium phosphate (TCP) crystals and PLA at varying loading levels of TCP (0, 5, 10, and 20 wt%) and assessed the composite scaffolds ability to induce proliferation and osteogenic differentiation of ASCs* in vitro*. In this study they were able to show that ASCs were able to adhere, proliferate, and osteogenically differentiate on all scaffolds; however there was a significant increase in the amount of cell-mediated mineralization in the highest TCP compared to the lowest TCP which suggest that the biochemical nature of the scaffold can accelerate and induce the overall osteogenic differentiation of ASCs [114]. In another study* in vivo*, they implanted PLGA/HA nanocomposite compared to PLGA scaffold only in a critical size defects in rat skulls where they found significant new bone formation with PLGA/HA in contrast with PLGA scaffold alone that had nearly no new bone formation. They concluded that the direct contact of cells with HA nanoparticles may stimulate osteogenesis [115]. There have been also some reports about other materials used and their effects on stem cells nonetheless there have not been any consensus on which nanoparticle/polymer composite is superior [97]. However the investigations that have been done till now with nanocomposite scaffolds demonstrate that such technology should be considered for bone tissue engineering application, as it will help and accelerate the differentiation and proliferation of ASCs into the osteogenic lineage leading to bone regeneration.

## 10. Growth Factors

**Figure 4:**
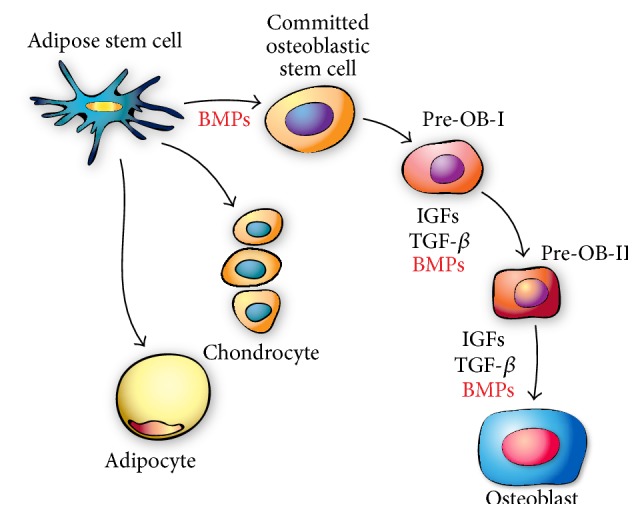
Schematic representation of BMP's effect on the differentiation of adipose stem cells into osteoblast.

Growth factors are usually used to enhance cell proliferation and differentiation of MSCs cells into osteogenic lineage. Multiple osteogenic growth factors were able to produce highly purified bioactive cytokines in large quantities, suitable for both cell culture and for* in vivo* applications. BMPs are considered to be the most promising osteogenic growth factors in stimulating bone formation and the most studied ones. They have been shown to be significantly involved with bone regeneration in fracture healing and DO as they trigger a cascade of events that lead to osteogenesis, chondrogenesis, angiogenesis, and upregulation of numerous growth factors [14]. They belong to the TGF-*β* superfamily that acts on many different systems including bone. BMPs are considered to be one of the most powerful osteogenic growth factor and the only osteoinductive ones that can act on undifferentiated MSCs early on (Figure 4) [116–118].

## 11. Stem Cells

Stem cells are undifferentiated progenitor cells that are capable of both self-renewal and multilineage differentiation [119]. They are classified into two categories, depending on their origin, the embryonic stem cells (ESCs) and the adult stem cells. The ESCs are derived from the inner cell mass of blastocyst-stage embryos [120], while the adult stem cells are derived from differentiated postnatal tissues and these are believed to be intimately involved in tissue/organ regeneration and repair during injury and ageing [121]. Only ESCs are considered to be pluripotent, since they are capable of giving rise to differentiated cell lineages of all three embryonic germ layers: endoderm, mesoderm, and ectoderm [122]. However their use is very limited by ethical, legal, and political concerns. On the other hand, adult stem cells are considered to be multipotent since they have lower degree of plasticity. Previously, it was believed that adult stem cells were only capable of differentiating into lineages that are characteristic of the tissue or organ from which they originated. However, recent evidence suggests that adult stem cells could possess a much higher degree of plasticity than previously thought [123, 124].

## 12. Mesenchymal Stem Cells

MSCs were initially discovered in bone marrow, after which they were isolated and characterized from several adult and fetal tissues, including adipose tissue, dermis, periosteum, umbilical cord blood, placenta and amniotic fluid, and synovial fluid [93, 125–129]. A set of minimal criteria were put in place by the international society for cellular therapy (ISCT) in order to be able to label a cell as MSC: cells must be plastic adherent when they are maintained in standard culture conditions, and they must express CD105, CD73, and CD90, lack the expression of CD45, CD34, CD14, CD11b, CD79*α*, or CD19 and HLA-DR surface molecules, and be capable of differentiating into osteoblasts, adipocytes, and chondroblasts* in vitro* [130]. MSCs have significant therapeutic potentials that can be applied to multiple disciplines especially where MSCs show low immunogenicity [131–133] and can differentiate into osteoblasts, chondroblasts, and adipocytes [134–136].

## 13. Bone Marrow Derived Mesenchymal Stem Cells (BM-MSCs)

MSCs were originally isolated from bone marrow by Friedenstein et al. [137], which have been considered the main source for MSCs for long time [138]. Bone marrow aspirates are collected from the iliac crest and MSCs are isolated and then cultivated. However, this is a painful and invasive procedure that often results in low yield of MSCs, especially that MSCs are present in relatively small quantities in the bone marrow and constitute about 0.001%–0.01% of the total marrow nucleated cells. Moreover, their ability to proliferate and differentiate declines after extensive passage or with age [93, 125, 138]. These disadvantages urged scientists to search for other sources of MSCs including adipose tissue.

## 14. Adipose Derived Mesenchymal Stem Cells

**Figure 5:**
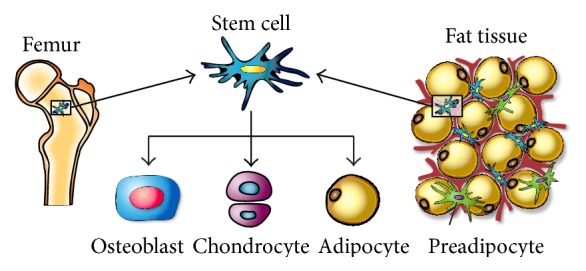
Schematic representation of stem cell isolation sites. This schematic shows that adult stem cells can be found in both bone marrow and adipose tissue. Both BM-MSC and ASC are capable of differentiating into the same three lineages' osteoblast, chondrocyte, and adipocyte.

ASCs where first isolated from human adipose tissue in 1976 [139], but they were not identified and characterized until 2001 [94]. Zuk et al. studied extensively the isolated ASCs from human lipoaspirates, and they showed that one gram of adipose tissue yields approximately 5 × 10^3^ stem cells, which is 500-fold greater than the number of MSCs in one gram of bone marrow indicating that adipose tissues are rich with MSCs and can be used as a promising alternative source for BM-MSC [140–143]. Over the last few years, several reports have shown that ASCs possess several advantages when compared to BM-MSCs. First, ASCs are readily available in large quantities, almost unlimited [141, 144], and can be retrieved in high volumes of cellular population with less invasive methods such as liposuction aspirates or subcutaneous adipose tissue fragments [94]. Moreover, ASCs can easily be expanded* in vitro*, have an extensive self-renewal capacity [95], and are easily isolated in a laboratory setting by differential sedimentation [94, 145, 146]. ASCs like BM-MSCs can differentiate into various cells, including adipocytes, osteoblasts, and chondrocytes (Figure 5) [134, 147].

## 15. Isolation of Mesenchymal Stem Cells from Adipose Tissue

Adipose tissue is comprised of adipocytes and a heterogeneous set of cell populations including endothelial cells, endothelial progenitor cells, pericytes, and erythrocytes that surround and support them, which upon isolation are termed the stromal vascular fraction (SVF) [142, 148, 149]. In order to isolate ASCs, adipose cells are harvested and then minced and digested by collagenase type II [145]. Then the SVF is separated by centrifugation as it has a higher density than the adipocytes [141, 145]. Later on, ASCs are isolated from the SVF by plastic adherence in culture, which can easily be cultured and expanded* in vitro* [145, 149, 150]. Moreover, isolated ASCs can be cryopreserved in a media of serum and dimethyl sulfoxide without losing their ability to differentiate and proliferate [151].

## 16. Adipose Stem Cells Immunophenotype

In order to be able to characterize ASCs and confirm that they abide with the ISCT guidelines, flow cytometry is used to determine the presence of specific cell surface markers. Since ASCs are not of homogenous population, there have been multiple attempts to find a unique single marker however that is still yet to be identified [95, 135, 152]. ASCs do express the typical MSCs markers such as CD13, CD29, CD44, CD63, CD73, CD90, and CD105, and ASCs show negative expression of for hematopoietic antigens such as CD14, CD31, CD45, CD 34, CD144, and HLA-DR [141, 142, 149, 153]. Some other markers are present at the beginning but do disappear or are lost during the different passaging while other will increase, as passaging will select more homogenous population with more homogenous cell surface markers [153–155]. Moreover, ASCs population included four groups with their own cell surface markers. The main two groups are preadipocytes cells, which represent 67.7% of the population and express CD31^−^/CD34^+^ surface markers, and premature endothelial cells which represent 5.2% and express CD31^+^/CD34^+^. The other two groups represent less than 1% and they are pericytes cells, CD146^+^/CD31^−^/CD34^−^, and the mature endothelial cells, CD31^+^/CD34^−^ [152, 156, 157]. ASCs have the ability to secrete growth factors such as FGF, VEGF, IGF, and TGF-*β*1 that stimulate and help tissue regeneration [158, 159]. Also, they are sensitive to different growth factors such FGF, PDGF, and VEGF [160]. This rendered ASCs ideal candidate for tissue engineering and bone regeneration.

## 17. Techniques for Direct ASCs Differentiation

### 17.1. Culture Milieu for ASCs Differentiation* In Vitro*

An important characteristic of ASCs is their ability to differentiation into multiple lineages, in particularly chondrocytes, osteocytes, and adipocytes, when the correct conditions are provided [123, 135, 146, 149]. The induction of ASCs differentiation* in vitro* is achieved by culturing ASCs in specific media [145].  Table 1 shows the different medias used for the induction of adipocytes, osteocytes, and chondrocytes.

### 17.2. The Use of Physical Stimuli to Differentiate MSCs into Osteoblasts

**Figure 6:**
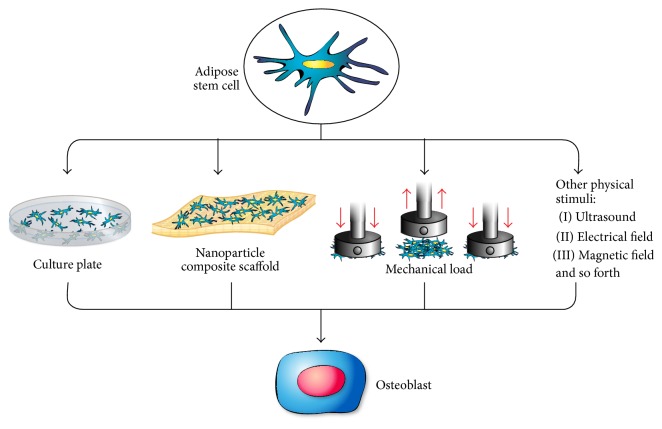
Schematic representation of different stimulating factors that can affect the differentiation of adipose stem cells into osteoblast.

Another less well-explored practice to differentiate stem cells is the application of physical stimuli including mechanical forces, magnetic, and electrical fields (Figure 6). Mechanical load has been identified as an essential factor in the development and maintenance of bone architecture and integrity [166, 167]. Increasing mechanical load leads to increase in bone mass by stimulating bone formation and inhibiting bone resorption through the activation of osteoblast cells and decreasing the activity of osteoclasts cells [168]. The process by which cells convert physical stimuli into biochemical response is called mechanotransduction [37, 169]. Mechanotransduction of stem cells is a very complex process that involves multiple signaling pathways that are not fully understood yet [167]. There have been few studies done to study the effect of mechanical stimuli on MSCs* in vitro* [170–174] and in animal models [175, 176], mainly on BM-MSCs [167]. Kapur et al. demonstrated the existence of multiple signaling pathways for stimulating osteoblast proliferation and differentiation from MSCs in response to mechanical stimuli. Mechanical stimuli can be applied either in the form of cyclic stretch [173, 177, 178] or in the form of fluid shear flow [179–182]. Duncan and Turner were able to show that shear stress causes primary and clonal osteoblast-like cells to proliferate and differentiate leading to increase in bone formation [183]. Also, Yoshikawa et al. were able to show that mechanical stimulation promotes the differentiation of osteogenic cells and enhances bone formation* in vitro* [172]. Moreover Altman et al. were able to show the differentiation of MSCs into osteogenic lineage under mechanical stimulus alone and without any exogenous growth factors [170]. Another source of physical stimuli is the application of electrical field that was shown to stimulate osteogenic differentiation from stem cells [184, 185]. The effect of pulsating electrical fields on osteogenesis has been studied in both animal models and* in vitro* studies and showed that it enhances stem cell signaling pathways and differentiation by modulating intracellular calcium signaling and augmenting tissue-specific markers [186, 187]. Recently, Hammerick was able to demonstrate an enhancement in early osteogenic markers in mouse ASCs using electrical field via ionic salt bridges; however there was no change in terminal differentiation [188]. Also, McCullen et al. studied the effect of sinusoidal alternating current electric fields on ASCs and demonstrated significant increase in intracellular calcium [189]. Other modalities are currently being investigated including magnetic fields [190, 191], ultrasound [192, 193], and laser irradiation [194, 195]. All these mechanical stimuli provide practical and promising methods to accelerate and facilitate the differentiation and proliferation of MSCs into osteogenic lineage cells that would be extremely significant in bone regeneration and tissue engineering.

## 18. BM-MSCs versus ASCs

Few studies were conducted to compare the characteristic differences between BM-MSCs and ASCs. Rebelatto et al. [134] have shown that both BM-MSCs and ASCs are morphologically and immunophenotypically similar expressing CD44, CD105, CD90, and CD73 and not expressing CD34, CD45, and HLA-DR. Moreover, both can differentiate into tissues of mesodermal and nonmesodermal origins. Strioga et al. compared the ontology and biology and preclinical and clinical application of ASCs and BM-MSCs [92]. They showed that both BM-MSCs and ASCs share many biological characteristics. However, some differences in their immunophenotype, differentiation potential, transcriptome, proteome, and immunomodulatory activity do exist and these differences may represent specific features of BM-MSCs and ASCs and inherent heterogeneity, simply due to the different isolation and culture protocols [92]. Nevertheless, ASCs are as effective as BM-MSCs in clinical applications. In fact ASCs are gaining the upper hand in clinical translational setting and stem cell-based tissue engineering.  Table 2 summarizes the major differences between BM-MSCs and ASCs.

## 19. Clinical Applications of ASCs in Bone Regeneration and DO

Bone regeneration represents a complex physiological and biological process that involves multiple cells recruitment and various signaling molecules and pathways that repair and regenerate bone in response to injury. In order for this complex mechanism to maximize its efficiency there are some requirements that have been identified including osteogenic cells, osteoconductive systems, and osteoinductive growth factors. This triangle concept represents an emerging state-of-the-art science and bone tissue engineering. This evolutionary science aims to repair a failing bone organ [3, 196].

However Giannoudis et al. have took this concept and modified into the diamond concept, which acknowledges the importance of implanting osteogenic cells in osteoconductive scaffolds and adding the essential growth factor in order to promote bone regeneration [197]. However, they emphasized the importance of two other factors that need to be addressed prior to any implantation or intervention. These factors are the optimization of the nonunion bed of the host in terms of adequate vascularization and the presence of adequate mechanical stability that will provide an appropriate environment for the cells to interact with the scaffold and growth factors in order to promote successful osteogenesis [198, 199]. For the purpose of this paper we have briefly discussed osteogenic cells including ASCs, scaffolds, and growth factors. The implication of ASCs in bone regeneration and DO will be summarized in the following paragraph.

Table 3 represents a summary of all the recent studies performed on ASCs in cases of DO and bone CSDs with the exception of maxillary/mandibular studies.

Liu et al. were the first to show that allogeneic ASCs combined with coral scaffolds are able to regenerate bone in critical-size defect model [204]. Cowan et al. introduced ASCs with PGLA scaffolds into CSD of mouse calvarial and had a significant increase in intramembranous bone formation by the end of two weeks and a complete bridging by 12 weeks without using any growth factors [205]. One study combined the benefits of using growth factors like BMP-2 with ASCs, where bone regeneration in calvarial CSD was enhanced [214]. Di Bella et al. reported that isolated autologous ASCs from rabbits implanted with osteoinduction fibronectin-coated porous cylindrical scaffolds improved bone regeneration in a critical-sized skull defect of rabbits [206]. On the clinical side, Lendeckel and colleagues described in a case report the use of autologous ASCs, bone graft, and fibrin glue all isolated from the patients tissue which were combined to treat cranial CSD in a young girl [207]. Three-month follow-up CT scan showed almost complete calvarial healing. All the previously mentioned studies concluded that ASCs can differentiate into osteoblasts with the capacity to regenerate bone and heal CSDs, this indicates that ASCs can be used as an alternative to bone graft in treating bone defects.

Studies describing the use of BM-MSCs or ASCs during DO are scarce in the literature. BM-MSCs were shown to accelerate new bone formation in DO models with shortened consolidation period [215]. Nomura et al. introduced ASCs mixed with collagen gels in rats after performing DO [208]. The research group observed an increase in bone formation in the ASC-Collagen injected rats compared to the control, and analysis of the formed callus showed both osteogenic differentiation and secretion of growth factors, which proves that ASCs promoted the formation of new bone. The therapeutic potentials of ASCs in tibial defects managed by DO were investigated in rabbits [209]. Radiologic analyses showed an increase in callus density, with increased ossification rate, in rabbits treated with osteoblast differentiated-ASCs compared to the control group treated with undifferentiated-ASC; they concluded that osteoblasts-differentiated ASCs shorten the consolidation period of DO [209]. In conclusion using ASCs instead of BM-MSCs ensure the availability of stem cells in abundance through a minimal invasive method without imposing any morbidity to the donor. Moreover, ASCs ability to regenerate bone in DO will accelerate the process and decrease the consolidation phase leading to an early removal of the fixator, which in return will decrease complications associated with DO such as infection, nonunion, psychological, and financial burden.

## 20. Perspectives and Challenges

Large bone defects continue to pose a formidable challenge to healing physicians. DO has been a very successful technique that is being used worldwide to treat multiple orthopedic and craniofacial complex conditions. However, as mentioned before, one major limitation is the long time the fixator needs to be kept in place until consolidation is done. Multiple methods and modalities have been used to accelerate the consolidation phase including the application of ASCs. As discussed above, ASCs fulfill most of the requirements needed for tissue engineering as they are available in an abundant quantities, can supply large number of cells, are easily accessible, have low immunogenicity, and are able to differentiate into multiple lineages and easy to isolate and expand* in vitro* [144]. ASCs have the potential to be used in the treatment of acute and chronic musculoskeletal disorders and other conditions. The Food and Drug Administration needs to approve the use of ASCs in bone regeneration and DO before it can be considered as a standard treatment. This will require significant preclinical research and development, some of which is outlined below.

ASCs are usually expanded and induced into osteogenic lineage using fetal bovine serum (FBS) medium. FBS medium is an animal derived product that can, theoretically, cause transmission of prions and bacterial or viral infections, even though it is very small [216] and increases the risk of immune reactions in the host to the xenogeneic materials used [217]. In order to overcome this problem, FBS free defined medium needs to be developed, optimized, and standardized. Few studies have already compared the use of human autologous serum to FBS on both ASCs and BM-MSCs and showed that human autologous serum produces comparable morphology, immunophenotype, and proliferation and differentiation capacity to FBS [148, 217–220]. Another alternative is allogeneic human serum that also showed promising results [221]. Therefore establishing and optimizing a safe and rapid expansion protocol for ASCs based on xeno-free culture are essential for cell-based therapies such as tissue engineering and regenerative medicine.

Also well-defined protocols for osteogenic differentiation from ASCs are needed for cell-based therapy. As discussed before there are multiple studies that have been done on this topic; however there has not been any consensus yet. Osteogenic differentiation may be enhanced if various techniques were combined together instead of being used alone. Therefore, combining the usually used culture media and supplementing it with growth factors such as BMPs and then allowing it to undergo some kind of physical stimuli might provide the optimal conditions required for osteogenic differentiation. This has not been studied enough yet but hopefully will be in the near future.

Another important area that needs to be clarified before taking any clinical testing is the potential tumorigenic feature of ASCs. There is a significant controversy in the current literature about the effect of ASCs on tumor growth as some studies showed that ASCs may favor the growth of tumor cells while other studies contradicted these results [222–225]. Yu et al. have shown that coinjection of human ASCs (hASCs) with tumor cells into BALB/c nude mice had increased the tumor cell viability* in vivo* and reduced the apoptotic cell death therefore favoring tumor growth* in vivo* [225]. Muehlberg et al. have also shown that mouse ASCs cause a significant rapid growth in cancer when added to murine breast cancer cell line. Moreover they showed that ASCs play an important role in tumor metastasis [223]. On the other hand, Cousin and his colleagues have shown that ASCs strongly inhibit pancreatic ductal adenocarcinoma proliferation both* in vitro* and* in vivo*. Moreover, ASCs induce tumor cell death [224]. Also Grisendi and colleagues have shown that ASCs can be used to support tumor necrosis factor related apoptosis-inducing ligand (TRAIL), which is a proapoptotic ligand that induces apoptosis in a variety of human cancers but not normal cells [226]. Hence, the literature is contradictory on the implication of ASCs in tumor growth and that can be partially explained by the different protocols used both* in vivo* and* in vitro*, which makes it hard to compare them. As in some studies, they combined ASCs injection with tumor cells while others did not. Moreover, different sources of ASCs were used including human and mouse ASCs and different types of cancers cells were investigated. To our knowledge, there are no studies which have been done to investigate the effect of ASCs on bone cancer. Thus, it is necessary to conduct more studies with consensual protocols to study the tumorigenic feature of ASCs and the long-term safety of using this technique before ASCs can be used as therapeutic tools in regenerative medicine and DO.

**Figure 7:**
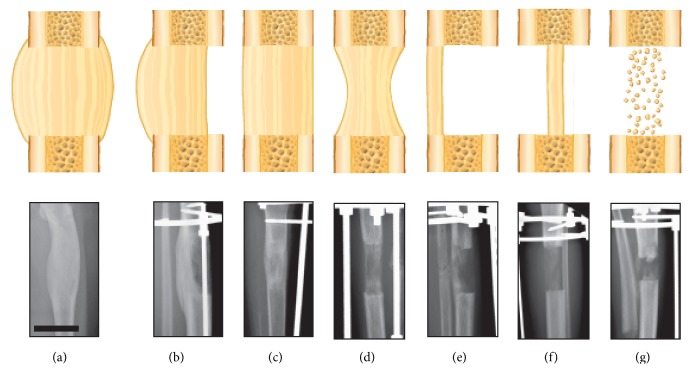
Representation of callus classification. Illustration and radiographs demonstrate the modified Li classification of callus shape in distraction osteogenesis. (a) New bone formation in the distraction gap extends beyond the outer borders of the cortical bone. (b) New bone formation toward one side of the distraction gap with extension beyond the outer borders of the adjacent cortical bone. (c) New bone formation within the distraction gap with margins parallel to the adjacent cortical bone. (d) Biconcave-shaped new bone formation within the distraction gap. (e) New bone formation limited to one side of the bone without extension beyond the outer borders of the cortical bone. (f) New bone formation in the center with limited new bone regeneration in the lateral portion of the distraction gap. (g) Only speckled bone formation is present (bar scale = 5 cm).

Another area of interest is the use of ASCs in DO. To date the scarce executed studies discussing the use of ASCs in DO did not provide a guideline with precise stepwise protocol of application of ASCs in DO model. From a therapeutic point of view, the use of ASCs should be carefully described according to the type of bone defect and callus shape. Donnan et al. classified callus depending on the shape, polarity, and consistency of callus regeneration [227]. Then Halvorsen et al. [162] described another classification based on callus shape and radiographic features which also was not used clinically due to its complexity. Finally Hamdy and McCarthy [228] developed a simplified version of Li classification that included seven callus shapes.  Figure 7 shows in detail the classified callus shapes, and Figures 7(a) through 7(c) show satisfactory regenerated bone that should heal without squeal; therefore no addition procedures are needed.  Figure 7(d) suggests that the rate of distraction is too fast, which can be easily corrected. However, Figures 7(e) through 7(g) are examples of poorly formed regeneration that may require enhancement before fixator removal to minimize complications. In order to improve the treatment outcome in these situations, the potential use of ASCs in bone engineering should be explored. Theoretically, in cases of partial/complete absence of bone formation in DO, the local administration of nanofibrous scaffolds with ASCs should be able to enhance and stimulate bone regeneration (Figures 7(e) and 7(f)). On the other hand nanocomposite scaffolds should be used in combination with ASCs to provide mechanical and biological support when there is suboptimal callus formation in DO (Figure 7(g)). To the best of our knowledge there have not been any previous studies investigating this promising strategies in the context of ASCs and DO.

Optimizing the concentrations of ASCs, mode of delivery and the time of application need to be extensively investigated in order to use this technology in DO and CSDs. It is important to validate if ASCs should be injected at the beginning, midway, or the end of distraction as this can have different impact on callus formation, the efficiency of ASCs proliferation, and differentiation. Moreover, the optimal concentration was never investigated before and such studies will be important to standardize a reproducible controlled guideline, to be used in clinical translational approaches.

Tissue engineering on bone regeneration as mentioned above requires scaffolds, growth factors and osteogenic cells. In DO the application of exogenic biological agents including growth factors has been a very-well documented approach to accelerate bone regeneration; however there are still some limitations such as short-half life, rapid clearance, and safety concerns [72]. Therefore developing an effective delivery system and combining them with stem cells are required. Nanobiomaterials have been used to convey growth factors in DO before [229], since they can achieve more effective and controlled release of the growth factors. Moreover, they can add strength to the mechanical properties of scaffolds and provide an environment that resembles the extracellular matrix of bone hence, promoting ASCs proliferation and differentiation into osteoblasts. Therefore, choosing the right nanocomposite scaffold is essential for the successful differentiation and proliferation of ASCs in DO. Further research is then needed to answer all these questions including when to inject stem cells in DO and when to inject nanocomposite scaffolds and which material is optimal in inducing osteogenic differentiation in DO? Which growth factor and delivery system are the optimal one to be used with stem cells for osteogenic induction? All these questions should be addressed in order to optimize the use of ASCs in bone regeneration and DO. Well-designed preclinical and translational studies to establish the safe and efficacious use of cell-therapies to enhance bone regeneration are needed.

## Figures and Tables

**Table 1:** Required supplements to induce differentiation of ASC into different lineages.

Cell lineage	Serum/media	Supplement required	Lineage characterization	Histologic/immunohistochemistry assay
Adipocytes [144, 149, 161]	(1) DMEM (2) 10% FBS	(1) Isobutylmethylxanthine (2) Insulin (3) Dexamethasone (4) Indomethacin	(1) Lipid accumulation	(1) Oil red O stain (2) Sudan III stain

Osteocytes [144, 149, 162]	(1) DMEM (2) 10% FBS	(1) 1,25-Dihydroxyvitamin (2) *β*-glycerophosphate (3) Ascorbate-2-phosphate (4) Dexamethasone	(1) Alkaline phosphatase activity (2) Production of calcified matrix	(1) Alizarin red stain (2) Von Kossa stain

Chondrocyte [163–165]	(1) DMEM (2) 1% FBS	(1) TGF-*β*1 (2) Insulin (3) Dexamethasone (4) Ascorbate-2-phosphate (5) BMP-6	(1) Sulfated proteoglycan rich matrix (2) Synthesis of collagen II	(1) Alcian blue stain (2) Collagen type II monoclonal antibodies

DMEM: Dulbecco's Modified Eagle's Medium, FBS: fetal bovine serum, BMP-2: bone morphogenetic protein-2, BMP-6: bone morphogenetic protein-6, and TGF-*β*1: tissue growth factor.

**Table 2:** Difference between BM-MSC and ASC.

Characteristics	BM-MSC	ASC
Stem cells isolation procedure [38, 41]	Invasive, complex	Noninvasive, Simple

The availability of stem cells in a given volume of BM aspirates or liposuction aspirate [41, 42]	Low	Abundant

Effect of donor's age on stem cell differentiation capabilities [36]	Decrease	Same

Site of collection [38, 41]	Iliac crest	Any fat tissue

Quantity available [41, 42]	Limited	Infinite

Differentiation [27, 28]	Adipocytes, osteocytes, chondrocytes	Adipocytes, osteocytes, chondrocytes

Immunogenicity [26]	Low	Low

**Table 3:** Overview of the studies performed on bone regeneration and distraction osteogenesis using ASC.

Author	Cell type	Scaffold	Model	Observation
ASC's application with scaffolds, without the use of growth factors				

Yoon et al. [200]	ASC & d-ASC	PLGA	Calvarial CSD in rats	d-ASCs with PGLA have better bone regeneration capability in CSD than constructs with ASC alone

Cui et al. [201]	ASC	Coral	Calvarial CSD in dogs	Bone was almost completely restored in the CSD, when ASCs were applied. Minimal bone formation with fibroid tissues was observed in the control group

Carvalho et al. [202]	ASC	SPCL	Calvarial CSD in mice	Nondifferentiated human ASCs enhance ossification of nonhealing mice CSD

Schubert et al. [203]	d- ASC	3D osteogenic ASC	(1) Four-level spinal fusion in pigs (2) Femur CSD in pigs	In a spine fusion model, applying 3D d-ASC demonstrated a significant increase in bone formation In the femoral CSD model, the 3D d-ASC achieved new bone formation and fusion in a poorly vascularized fibrotic environment

Liu et al. [204]	Al- ASCs, Au-ASC	Coral	Cranial CSD in dogs	Allo-ASC transplantation did not induce a systemic immune response and was able to repair the cranial CSDs in an analogous way to that of the autologous cells

Cowan et al. [205]	ASC	PGLA	Calvarial CSD in mice	ASC showed a significant intramembranous bone formation by 2 weeks and complete bridging by 12 weeks without any additives

Di Bella et al. [206]	ASC, d-ASC	PLA & FPLA	Skull CSD in rabbits	(1) FPLA as a fibronectin-coated scaffold promotes bone formation more than using PLA alone (2) d-ASCs combined with FPLA enhance bone formation significantly when compared with ASC alone

Lendeckel et al. [207]	ASC	Fibrin glue & bone graft	Cranial CSD in a 7-year-old girl	Complete calvarial healing after 3 months

Nomura et al. [208]	ASC	Collagen gel	DO femur in rats	ASC promoted bone formation in the distracted callus and shortening the consolidation phase

Sunay et al. [209]	ASC, d-ASC		DO tibia in rabbits	d-ASC showed increase in the callus density and the ossification rate compared to the undifferentiated ASC. The quality of bone formed within the callus was significantly enhanced. Use of d-ASC can shorten the consolidation period of distraction osteogenesis

Arrigoni et al. [210]	ASC	HA	Tibia CSD in rabbits	ASCs-HA constructs improved bone healing significantly, when compared to using scaffold alone

Cheng et al. [211]	ASC	DBM	Calvarial CSD in rabbits	New bone formation was documented in bone defects transplanted with DBM-ASCs composites

ASC's application with scaffolds, with the use of growth factors like BMP2 and TGF (*β*3)				

Lin et al. [212]	BMP2 expressing ASC and TGF (*β*3)	PLGA or gelatin sponge	Calvarial CSD in rabbits	Gelatin sponges and apatite coated PLGA were compared as scaffolds. Gelatin scaffold stimulated the bone healing more than apatite coated PLGA, regardless of BMP2 or TGF-*β*3 expression. The ASCs/gelatin expressing BMP2 triggered better bone healing than ASCs/gelatin expressing TGF-*β*3

Peterson et al. [213]	HPLA, with BMP-2 carrying adenovirus	CCC	Femur CSD in rats	HPLA cells genetically modified by adenoviruses, overexpressing BMP-2, can induce bone formation *in vivo* and heal CSD in rat femurs

Levi et al. [214]	Human ASC, with BMP-2	PGLA	Calvarial CSD in mice	(1) Human ASCs ossify CSD without the need for predifferentiation (2) rBMP-2 was observed to increase human ASC osteogenesis *in vitro* and osseous healing *in vivo*

GF:growth factor, PLGA: polylactide-co-glycolic acid, d-ASC: differentiated ASC, PLA: polylactic acid, SPCL: wet-spun starch polycaprolactone, Allo-ASC: allogeneic ASC, Au-ASC: autologous ASC, FPLA: fibronectin-treated PLA, HPLA: human processed lipoaspirate, CCC: collagen-ceramic carrier, HA: hydroxyapatite, and DBM: demineralized bone matrix.
